# Experimental and computational investigation of a DNA-shielded 3D metal–organic framework for the prompt dual sensing of Ag^+^ and S^2−^†

**DOI:** 10.1039/c9ra02028d

**Published:** 2019-05-17

**Authors:** Shao-Lan Cai, Zi-Chuan Yang, Ke-Yang Wu, Cheng Fan, Ling-Yan Zhai, Nai-Han Huang, Rong-Tian Li, Wen-Jun Duan, Jin-Xiang Chen

**Affiliations:** Guangdong Provincial Key Laboratory of New Drug Screening, School of Pharmaceutical Sciences, Southern Medical University Guangzhou 510515 P. R. China jxchen@smu.edu.cn wjduan@smu.edu.cn +86-20-61648533

## Abstract

We herein report an efficient Ag^+^ and S^2−^ dual sensing scenario by a three-dimensional (3D) Cu-based metal–organic framework [Cu(Cdcbp)(bpea)]_*n*_ (MOF 1, H_3_CdcbpBr = 3-carboxyl-(3,5-dicarboxybenzyl)-pyridinium bromide, bpea = 1,2-di(4-pyridinyl)ethane) shielded with a 5-carboxytetramethylrhodamine (TAMRA)-labeled C-rich single-stranded DNA (ss-probe DNA, P-DNA) as a fluorescent probe. The formed MOF-DNA probe, denoted as P-DNA@1, is able to sequentially detect Ag^+^ and S^2−^ in one pot, with detection limits of 3.8 nM (for Ag^+^) and 5.5 nM (for S^2−^), which are much more lower than the allowable Ag^+^ (0.5 μM) and S^2−^ (0.6 μM) concentration in drinking water as regulated by World Health Organization (WHO). The detection method has been successfully applied to sense Ag^+^ and S^2−^ in domestic, lake, and mineral water with satisfactory recoveries ranging from 98.2 to 107.3%. The detection mechanism was further confirmed by molecular simulation studies.

## Abstract 2

We herein report an efficient Ag^+^ and S^2−^ dual sensing scenario by a three-dimensional Cu-based metal–organic framework shielded with a 5-carboxytetramethylrhodamine-labeled C-rich single-stranded DNA as a fluorescent probe.

## Graphical abstract

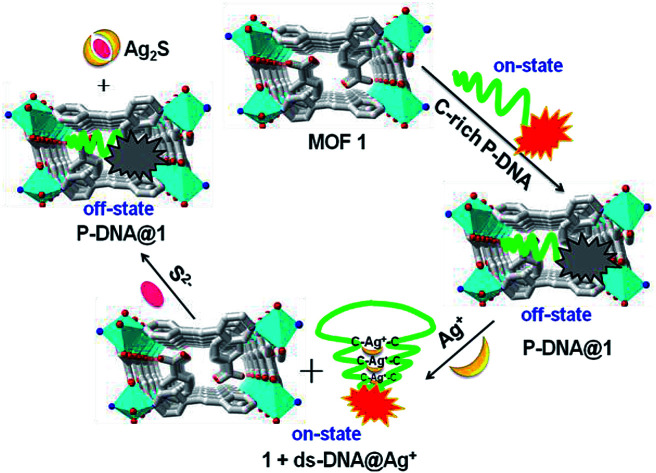

## Introduction

Silver ions (Ag^+^) are ubiquitous and widely applied in various fields including biomedicine, antibacterial manufacturing, *etc.*^1^ However, the biological accumulation of Ag^+^ raises serious concerns as a result of its relevance to multiple organ dysfunction syndrome, cytotoxicity, argyria and weakness of mitochondrial ability.^2^ Ag^+^ is accumulated *via* the water and food cycle, and thus a 0.5 μM concentration up-limit of Ag^+^ in drinking water is regulated by U.S. Environmental Protection Agency (US EPA) and World Health Organization (WHO).^3^

On the other hand, sulfide (S^2−^) possesses an important biological function.^4^ S^2−^ exhibits a strong affinity toward proton to give hydrogen sulfide (H_2_S), which is the third largest gas signal molecules. H_2_S takes part in the course of atherosclerosis, myocardial contraction, nerve transmission and regulation of insulin secretion.^5^ Moreover, high concentrations of H_2_S gives rise to diseases such as diabetes,^6^ hepatic sclerosis,^7^ Alzheimer's disease,^8^ and Down's syndrome.^9^

An equally important concern is the co-existence of Ag^+^ and S^2−^ that would result in the formation of Ag_2_S precipitates due to its extremely low solubility product constant of Ag_2_S (*K*_sp_ = 6.3 × 10^−50^, r.t.). Ag_2_S particle formation is harmful to eyes, skin and respiratory system.^10^ It would, therefore, be essential to develop a sensor that can simultaneously detect the presence of Ag^+^ and S^2−^. Some hazardous ions can be monitored by fluorescence-based chemosensors as a result of their rapid detection speed, simple procedure, and high sensitivity.^11^ Among them, metal–organic frameworks (MOFs), consist of metal ions and various organic ligands, have shown great potential in sensing metal ions and small molecules due to their unique advantages such as diversity, porosity, stability, amenability toward further functionalization.^12^ However, sensors by integrating MOFs and bio-related species, such as DNA, remains relatively rare.^13^

**Fig. 1:**
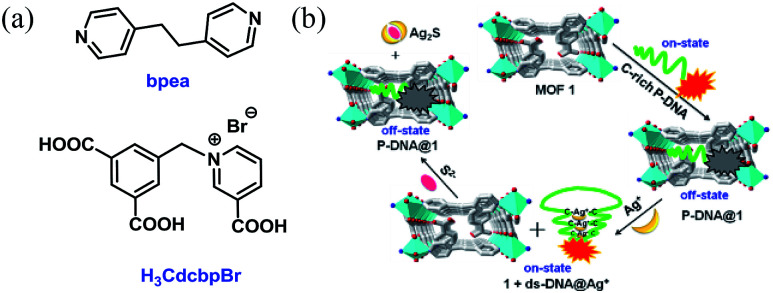
(a) The structures of bpea and H_3_CdcbpBr. (b) The detection mechanism of Ag^+^ and S^2−^ based on the hybrid of MOF 1 and C-rich P-DNA.

Herein, we report the construction of a sensing system by hybridizing a three-dimensional (3D) Cu-based MOF [Cu(Cdcbp)(bpea)]_*n*_ (MOF 1, H_3_CdcbpBr = 3-carboxyl-(3,5-dicarboxybenzyl)-pyridinium bromide, bpea = 1,2-di(4-pyridinyl)ethane, Fig. 1a) and a single-stranded, carboxytetramethylrhodamine (TAMRA)-labeled C-rich probe DNA (ss-DNA, P-DNA). The MOF and P-DNA are associated through π–π stacking, hydrogen bonding, and electrostatic interactions, and thereby quenches the TAMRA fluorescence of the latter (off-state) *via* a photo-induced electron transfer to eliminate the high background fluorescence (Fig. 1b).^14^ The formed MOF-DNA probe, denoted as P-DNA@1, is able to sense the presence of Ag^+^ through the formation of C–Ag^+^-C coordination bonds, yielding the double-stranded hairpin-like DNA, that is ds-DNA@Ag^+^ duplex.^15^ The much more rigid ds-DNA@Ag^+^ duplex formed is subsequently detached from the surface of MOF 1 with the recovery of the TAMRA fluorescence (on-state). When further adding S^2−^ to the above formed 1 + ds-DNA@Ag^+^ mixture, Ag^+^ was extracted from ds-DNA@Ag^+^ to form Ag_2_S precipitates and the ds-DNA unwinded and converts back to P-DNA, and resorbed by MOF 1 to quench the fluorescence again (off-state). Thus, the “off-on-off” fluorescent sensing system was successfully constructed to monitor Ag^+^ and S^2−^ in succession. The computational investigation revealed that P-DNA bounds to MOF 1 more tightly through multiple π–π stacking and hydrogen bonding interactions than ds-DNA@Ag^+^.

## Experimental

### General

IR spectra were recorded on a Nicolet MagNa-IR 550 infrared spectrometer. Elemental analyses for C, H, and N were carried out with an EA1110 CHNS elemental analyzer. Powder X-ray diffraction (PXRD) spectra were obtained with a Rigaku D/max2200/PC. The X-ray generated from a sealed Cu tube was monochromated by a graphite crystal and collimated by a 0.5 mm MONOCAP (*λ* Cu-Kα = 1.54178 Å). The tube voltage and current were 40 kV and 40 mA, respectively. Fluorescence spectra were measured on a PE LS-55 fluorescence spectrophotometer.

The synthesis of amphoteric tricarboxylic acid ligand-H_3_CdcbpBr was based on our reported procedure.^14*c*^ The TAMRA-labeled cytimidine-rich probe DNA sequence (P-DNA: 5′-TAMRA-ACCTCTTCTCTTCATTTTTCAACACAACACCG-3′) was purchased from Sangon Inc. and stored at −20 °C for use, and at −80 °C for long-term preservation. All the other reagents and solvents were obtained from commercial sources and used without further purification.

### Synthesis of [Cu(Cdcbp)(bpea)]_*n*_ (1)

Powder of H_3_CdcbpBr (764 mg, 2 mmol) was dispersed in distilled H_2_O (70 mL) by sonication and the pH adjusted to 7.0 with 0.1 M NaOH to give a clear solution. This is followed by slow dropwise addition of CuSO_4_·5H_2_O (391 mg, 2 mmol) dissolved in H_2_O (20 mL). The formed mixture was stirred for 30 min to give a clear blue solution. Subsequently, bpea (380 mg, 2 mmol) dissolved in DMF (5 mL) was slowly added and the mixture was shaken for a while to give a blue solution containing a small amount of blue precipitate. Upon filtration, the filtrate was sub-packaged in thick-walled pressure bottle and transferred to a programmed oven. The temperature of the oven was smoothly increased from r.t. to 100 °C in 1 h, maintained at 100 °C for 72 h, before finally cooled to r.t. within 48 h to give blue block crystals. The crystals obtained were collected by filtration and washed with diethyl ether and dried *in vacuo* (910 mg, 83%). Anal. calcd for C_27_H_21_N_3_O_6_Cu·H_2_O: C 57.35%, H 4.07%, N 7.43%; found: C 57.16%, H 3.98%, N 7.37%. IR (KBr disc, cm^−1^) *ν* 3324 (s), 3123 (m), 1635 (s), 1489 (m), 1378 (s), 1220 (m), 1154 (m), 1018 (w), 832 (m), 815 (m), 727 (m), 614 (m), 478 (m).

### X-ray crystal structure determination

Crystallographic measurements were made on a Bruker APEX II diffractometer by using graphite-monochromated Mo Kα (*λ* = 0.71073 Å) irradiation for MOF 1. The data were corrected for Lorentz and polarization effects with the SMART suite of programs and for absorption effects with SADABS.^16^ All crystal structures were solved by direct methods and refined on F^2^ by full-matrix least-squares techniques with SHELXTL-97 program.^17^ The location of the two hydrogen atoms on the coordinated water was suggested by Calc-OH program in the WinGX suite,^18^ the water molecules were then refined as a rigid model with their thermal parameters constrained to *U*_iso_(H) = 1.2*U*_eq_(O). Spatially delocalized electron density in the lattice was found but acceptable refinement results could not be obtained for this electron density. The solvent contribution was then modeled using SQUEEZE in Platon.^19^ A summary of the key crystallographic data for 1 is listed in Table S1.†

## Results and discussion

### Characterization of MOF 1

**Fig. 2:**
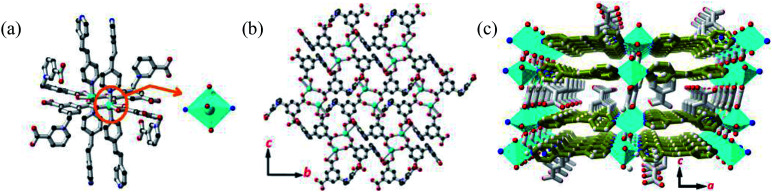
The structure of MOF 1. (a) The coordination environment of Cu^2+^ in MOF 1. (b) The 2D plane sheet structure constructed by the [Cu_2_(Cdcbp)_4_] units. (c) The 3D structure of MOF 1. Color codes: Cu (turquoise), O (red), N (blue), C (black) and bpea (olive green).

Single crystal X-ray diffraction analysis revealed that MOF 1 crystallizes in the monoclinic space group *C*2/*c* and the asymmetric unit consists one [Cu(Cdcbp)(bpea)] molecule. As shown in Fig. 2a, each two Cu(ii) ions are linked by a couple of μ–COO^−^ (η^1^:η^1^) groups from two Cdcbp ligands, and each Cu(ii) ion further coordinated one chelating carboxylate to form a [Cu_2_(Cdcbp)_4_] unit (Fig. 2a). Such unit extends to six equivalents to form a two-dimensional (2D) structure within the *bc* plane as shown in Fig. 2b. These adjacent 2D layers have their associated benzoate oriented face-to-face and approximate 13.4 Å apart, an ideal distance to further accommodate one bpea ligand (13.4 Å approximates the distance of one bpea ligand of *ca.* 9.4 Å and a pair of Cu–N bonds with *ca*. 2.0 Å for each) (Table S2†). The Cu–bpea association is aligned in the *a* direction, completes the octahedral coordination geometry of Cu^2+^ (Fig. 2a), and result in a 3D network of MOF 1 as shown in Fig. 2c.

### Ag^+^ detection with P-DNA@1 hybrid and S^2−^ detection with 1 + ds-DNA@Ag^+^ system

MOF 1 is water stable and its powder X-ray diffraction (PXRD) pattern indicated that the as-synthesized MOF 1 and its fresh powder immersed in Hepes buffer (pH = 6.5, 7.0, 7.4) for 24 h matched very well with the simulated one, suggesting its phase purity and buffer stability (Fig. S1†). The scanning electron microscopic (SEM) images of the fresh MOF 1 gave the size of 831.25 ± 80 nm, further indicating its phase homogeneity (Fig. S2†).

**Fig. 3:**
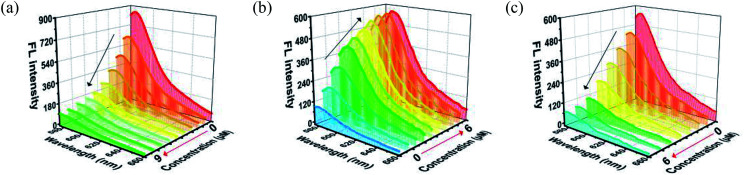
(a) The fluorescence quenching of the P-DNA (50 nM) incubated with different concentrations of MOF 1. (b) The fluorescence recovery of P-DNA@1 (50 nM/9.0 μM) sensing system towards different concentrations of Ag^+^. (c) The fluorescence quenching of 1 + ds-DNA@Ag^+^ (9.0 μM/50 nM/6.0 μM) sensing system towards various concentrations of S^2−^.

**Fig. 4:**
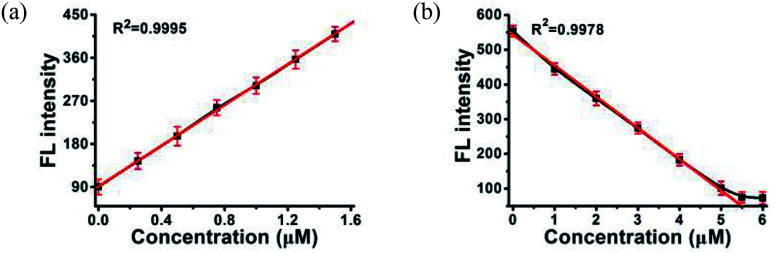
A linear relationship between the fluorescence intensity of (a) P-DNA@1 at 582 nm and the concentrations of Ag^+^, (b) 1 + ds-DNA@Ag^+^ at 582 nm and the concentrations S^2−^. Error bars represent the standard deviation for three measurements.

In MOF 1, the Cdcbp and bpea ligands contain conjugated π-electron system, Cu^2+^, and quaternary ammonium groups, all facilitate its binding with ss-P-DNA molecules through a variety of weak intermolecular interactions, such as π–π interactions and hydrogen bonding, and ultimately lead to fluorescence quenching.^20^ In order to verify our hypothesis, we first studied the stability of P-DNA in Hepes buffer at three different pH conditions (6.5, 7.0, 7.4) that approximate the physiological conditions. As shown in Fig. S3,† the emission profile is retained in these condition in 4 h. Then we studied the interaction of MOF 1 with P-DNA at these pH conditions to make the Ag^+^ sensor. As shown in Fig. 3a, S4a and S5a,† in all three conditions, the fluorescence intensity of P-DNA gradually decreased with the increasing concentration of MOF 1 up to 9.0 μM with a quenching efficiency (*Q*_E_, %) of 89.3% due to the formation of P-DNA@1 (*Q*_E_ = (*F*_0_ − *F*_M_)/*F*_0_ × 100%, wherein *F*_M_ and *F*_0_ are the fluorescent intensities at 582 nm in the presence and absence of MOF 1, respectively). Our second step involves the sensing of Ag^+^ using P-DNA@1 also at three different pH conditions (6.5, 7.0, 7.4). As shown in Fig. 3b, S4b and S5b,† in all three conditions, when Ag^+^ was added into the P-DNA@1 sensing system with gradually increasing concentrations, the fluorescence intensity recovered to saturation with a concentration of Ag^+^ from 0 to 6.0 μM. The recovery efficiency (*R*_E_) was 4.9 on the basis of *R*_E_ = (*F*_T_ − *F*_M_)/*F*_M_ (*F*_T_ and *F*_M_ are the fluorescence intensities at 582 nm with and without Ag^+^, respectively). The fluorescence recovery spectra exhibits a linear relationship with the concentration of Ag^+^ (Fig. 4a), and a linear equation of *Y* = 212.559*X* + 91.532 with a related coefficient *R*^2^ = 0.9995 can be derived. The detecting limit (LOD) of Ag^+^ was 3.8 nM calculated from 3*σ*/slope (*σ* = standard deviation for 10 blank samples), which is much lower than the reported materials, such as tetraphenyl ethylene (874 nM),^21^ carbon dots (320 nM),^22^ imidazole derivatives (101 nM),^23^ and comparable to gold nanoparticle (7.3 nM)^24^ and g-C_3_N_4_ nanosheets (4.2 nM) (Table S3†).^25^ Our third experiment concerns the sensing of S^2−^ using the above formed 1 + ds-DNA@Ag^+^ system at three different pH conditions (6.5, 7.0, 7.4). As shown in Fig. 3c, 4c and S5c,† in all three conditions, when various concentrations of S^2−^ were added to the 1 + ds-DNA@Ag^+^ system, the fluorescence intensity gradually decreased to stabilization with a maximum S^2−^ consumption of 6.0 μM (*Q*_E_ value being 86.9%). The fluorescence quenching spectra of 1 + ds-DNA@Ag^+^, as depicted in Fig. 4b, also showed good linearity between fluorescence intensity and S^2−^ concentration with the linear equation of *Y* = −89.855*X* + 544.153 (*R*^2^ = 0.9978), giving an LOD value of 5.5 nM for S^2−^. Such a value is much lower than some nanocomposite (138 nM),^26^ gold nanoparticles (80 nM),^27^ DNA@copper nanoparticles (80 nM),^28^ and nano Ag–carbon (27 nM),^29^ and comparable to g-C_3_N_4_ nanosheets (3.5 nM) (Table S4†).^25^ All the results indicate that the detection process was not disturbed by the variation of the pH values.

### The selectivity of the sensor

**Fig. 5:**
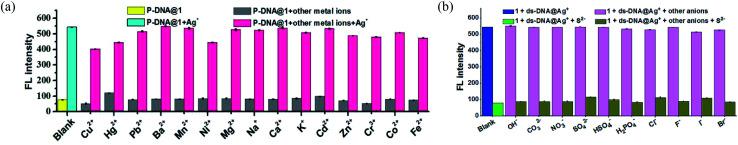
(a) The detection selectivity of Ag^+^ sensor (blank: P-DNA@1 (50 nM/9.0 μM); Ag^+^: 6.0 μM, other metal ions: 30 μM). (b) The detection selectivity of S^2−^ sensor (blank: 1 + ds-DNA@Ag^+^ (9.0 μM/50 nM/6.0 μM); S^2−^: 6.0 μM, other anions: 30 μM). Error bars represent the standard deviation for three measurements.

The anti-interference ability is another critical characteristic for the biosensors to ensure their practical application. Herein, the P-DNA was designed with specific bases that can pair with silver exclusively to form “C–Ag^+^–C”.^15^ To further confirm our hypothesis, we carried the following experiments. First, different kinds of metal ions were used as interferences, including Hg^2+^, Mn^2+^, Cd^2+^, Ca^2+^, Pb^2+^, Mg^2+^, Cu^2+^, Ba^2+^, Ni^2+^, K^+^, Na^+^, Zn^2+^, Cr^3+^, Co^2+^ and Fe^2+^ with a concentration of 30 μM. As depicted in Fig. 5a, the biosensor of P-DNA@1 showed no remarkable response to these interfering metal ions. Subsequently, we added Ag^+^ (6.0 μM) to each of the above metal ion solutions, and the fluorescence intensities increased dramatically in contrast with other interfering metal ions with 5-fold concentrations higher than that of Ag^+^. It is thus conclusive that the system had a high selectivity towards Ag^+^. Likewise, the specificity of the S^2−^ sensor was also evaluated by comparing its response to S^2−^ and other small anions (HSO_4_^−^, SO_4_^2−^, OH^−^, H_2_PO_4_^−^, CO_3_^2−^, NO_3_^−^, F^−^, Cl^−^, Br^−^ and I^−^) with a concentration of 30 μM (5-fold higher than S^2−^). As illustrated in Fig. 5b, there was no obvious fluorescence intensity changes induced by other anions while the fluorescence intensity declined significantly when S^2−^ (6.0 μM) was presented. Thus, the interference of the other small anions to the biosensor 1 + ds-DNA@Ag^+^ was also negligible.

**Fig. 6:**
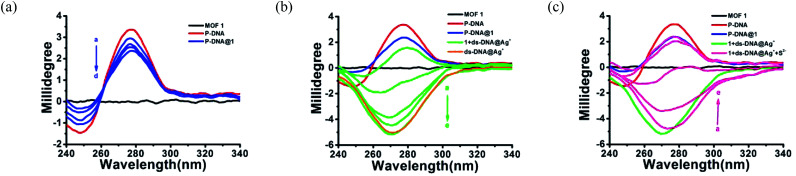
(a) The CD spectra of MOF 1 (150 μM) and P-DNA (5 μM) with different concentrations of MOF 1 (50, 100, 125, 150 μM, a–d). (b) The CD spectra of P-DNA@1 (5 μM/150 μM) with different concentrations of Ag^+^ (12, 24, 36, 48, 60 μM, a–e) and ds-DNA@Ag^+^ (5 μM/60 μM). (c) The CD spectra of 1 + ds-DNA@Ag^+^ (150 μM/5 μM/60 μM) with different concentrations of S^2−^ (20, 40, 60, 80, 100 μM, a–e).

In order to further clarify the detection process, the conformational changes of the P-DNA induced by Ag^+^ and S^2−^ were investigated by circular dichroism (CD) spectroscopy. Single-stranded P-DNA with a concentration of 5 μM exhibited a strong positive CD peak at 278 nm. As shown in Fig. 6a, the intensity of the peak gradually decreased with increased concentrations of MOF 1. MOF 1 fails to absorb all the P-DNA (5 μM) at its highest concentration of 150 μM, and only lead to partial disappearance of the CD peak of P-DNA. With an increased concentration of Ag^+^ in the P-DNA@1 sensing system, the intensity of positive peak weakened and ultimately disappeared, accompanied by the continuous intensity increase of the negative peak (Fig. 6b), attributable to the formation of a large amount of C–Ag^+^–C ds-DNA@Ag^+^ complex.^30^ Finally, with the addition of S^2−^ to the 1 + ds-DNA@Ag^+^ system, the negative peak disappeared and the positive peak appeared again, indicating that the ds-DNA@Ag^+^ unwind to release P-DNA (Fig. 6c).

### The detection mechanism

In order to elucidate the detection mechanism, the binding free energy difference (ΔΔ*G*) between reactions of MOF 1 with P-DNA (Δ*G*_P-DNA@MOF_) and MOF 1 with ds-DNA@Ag^+^ (Δ*G*_MOF+ds-DNA@Ag^+^_) is calculated. The Gibbs free energy calculations were simplified by calculating single point energies. The result (Table S5†) showed that ΔΔ*G* = −180.24 kcal mol^−1^ < 0, suggesting that P-DNA bounds to MOF 1 more tightly than ds-DNA@Ag^+^, corroborating the experimental observation.

**Fig. 7:**
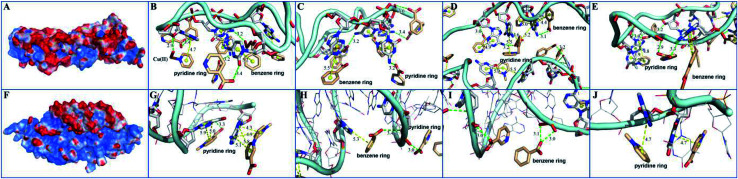
The interactions between MOF 1 and P-DNA and ds-DNA@Ag^+^: (A) and (F) showed the charge distributions where red area represented negative charges, blue positive, and white neutral; (B–E) and (G–J) showed the local binding modes for MOF 1 with P-DNA (B–E) and MOF 1 with ds-DNA@Ag^+^ (G–J), where MOF 1 was displayed by smudge lines and sticks, while P-DNA and ds-DNA@Ag^+^ by light blue lines and sticks. Aromatic ring centers were denoted by yellow spheres, π–π stacking, and hydrogen bonding interactions by yellow dashes and green dashes.

The electrostatic surface showed that the surface of MOF 1 was largely positive and P-DNA covered by a negative and neutral surface with the scattered positive area, which led to good electronic complement between MOF 1 and P-DNA. Single chain P-DNA exhibits a higher contact area with MOF 1 due to its structural flexibility (Fig. 7A), while double chain ds-DNA@Ag^+^ only has a small interface (Fig. 7F). MOF 1 bound to P-DNA mainly through 19 π–π stacking (Fig. 7B–E) between the aromatic rings in MOF 1 and nucleobases in P-DNA with the ring centroid distances in the range of 3.8–5.9 Å. This is in addition to 18 hydrogen bonding (Fig. 7B–E) between the nucleobases in P-DNA and carboxylate groups in MOF 1, with the donor–acceptor distances in the range of 2.9–3.4 Å. In sharp contrast, there are only 9 π–π stacking between the aromatic rings in MOF 1 and the nucleobases in ds-DNA@Ag^+^, and 5 hydrogen-bondings between oxygen/nitrogen atoms of the phosphate group, or the nucleobases in ds-DNA@Ag^+^ and carboxylate groups in MOF 1 (Fig. 7G–J), with the ring centroid distances in the range of 4.2–5.8 Å and donor–acceptor distances in the range of 2.8–3.5 Å.

### Detect Ag^+^ and S^2−^ in environmental water

To further demonstrate the applications of these two biosensors, the recovery efficiencies of Ag^+^ and S^2−^ were studied in three kinds of environmental water samples, including domestic water, lake water, and mineral water. The environmental water samples of 0.6 μM Ag^+^ and S^2−^ were prepared from a condensed stock solution of silver ion (10 mM) and sulfide ion (10 mM), respectively. Both Ag^+^ and S^2−^ in environmental water samples were measured as mentioned in the previous section. Each measurement was carried out three times repeatedly. As shown in Tables S6 and S7,† the recovery efficiencies of the Ag^+^ and S^2−^ were calculated to be in the range of 98.2–101.8% with standard deviations (RSD) ≤ 1.0% for Ag^+^, and from 99.0% to 107.3% with RSD ≤ 4% for S^2−^, respectively. These results verified the reliability and practicability of the proposed fluorescence sensor for the successive detection of Ag^+^ and S^2−^.

## Conclusions

In summary, a highly sensitive and selective fluorescence sensor was created based on the Cu-MOF shielded with fluorescence-labeled C-rich ss-P-DNA for the specific and successive detection of Ag^+^ and S^2−^ with a low detection limit of 3.8 nM and 5.5 nM, respectively, which were two orders of magnitudes lower than those allowed in drinking water. The uniqueness of the present probe arises from the use of C–C mismatched P-DNA sequences 5′-TAMRA-ACCTCTTCTCTTCATTTTTCAACACAACACCG-3′ that can exclusively recognize Ag^+^ to form C–Ag(i)–C duplex.^15^ The use of Cu-based MOF 1 as fluorescence quencher serves as an additional advantage as the Ag^+^ sensing process is against a dark background. The sensing events are stable at three different pH conditions (6.5, 7.0, 7.4) that mimicking physiological conditions, and are further transferrable to domestic, lake, and mineral waters, indicating the good adaptability of the present sensor. This discovery further inspired us to develop relevant sensors for the pairwise detection of other biologically relevant cations and anions, such as Hg^2+^ and S^2−^, Pb^2+^ and Br^−^, Fe^3+^ and ascorbic acid, and *etc.*, using a shared mechanism and beyond.

## Conflicts of interest

There are no conflicts to declare.

## Supplementary Material

RA-009-C9RA02028D-s001

RA-009-C9RA02028D-s002
