# Supplementary material for: Experimental and computational investigation of a DNA-shielded 3D metal–organic framework for the prompt dual sensing of Ag+ and S2−
Source: RSC Adv. 2019 May 17;9(27):15424–30. doi: 10.1039/c9ra02028d (PMC9064208; doi:10.1039/c9ra02028d)
Supplement: RA-009-C9RA02028D-s001 [file RA-009-C9RA02028D-s001.pdf]

Electronic Supplementary Material (ESI) for RSC Advances.
This journal is © The Royal Society of Chemistry 2019

Experimental and computational investigation of a DNA-shielded 3D metal−organic framework for the prompt dual sensing of Ag^+ and S^{2-}

Shao-Lan Cai, Zi-Chuan Yang, Ke-Yang Wu, Cheng Fan, Ling-Yan Zhai, Nai-Han Huang, Rong-Tian Li, Wen-Jun Duan* and Jin-Xiang Chen*

Ag^+ and S^{2-} detection experiments……………………………………………………..…........…S-2
Computational molecular simulation studies……………………………………...….…...S-3
Table S1 Crystallographic data for **1**…………………………………………………..……........S-4
Table S2 Selected bond distances (Å) and angles (º) for **1**…………………..………………….S-5
Table S3 The analytical performance of various Ag^+ sensors……..………………....................S-5
Table S4 Comparison of different sensing platforms for S^{2-} detection…...………......................S-5
Table S5 The single point energy results of P-DNA, P-DNA@**1**, ds-DNA@Ag^+ and **1** + ds-DNA@Ag^+...S-6
Table S6 Detection of Ag^+ in environmental water samples…………………………….........S-6
Table S7 Detection of S^{2-} in environmental water samples..S-6
Fig. S1 PXRD patterns of MOF **1** showing agreement among the simulated, as-synthesized and fresh powder of MOF **1** immerse in Hepes buffer (20 mM, pH = 6.5, 7.0, 7.4) for 24 h respectively......................…………………………………………………..……..…...…S-7
Fig. S2 The SEM image of MOF **1**...............………………...…………………….…….........S-7
Fig. S3 Comparison of the intensity of the emission peak (582 nm) of the P-DNA in 20 mM Hepes buffer ((pH = 6.5, 7.0, 7.4) in 4 h..................…………………...…………………….……...S-8
Fig. S4 (a) The fluorescence quenching of the P-DNA (50 nM) incubated with MOF **1** with increasing concentrations in Hepes buffer (pH 7.4, 20 mM). (b) The fluorescence recovery of P-DNA@**1** (50 nM/ 9 μM) sensor towards Ag^+ with different concentrations in Hepes buffer (pH 7.4, 20 mM). (c) The fluorescence quenching of **1** + ds-DNA@Ag^+ (9 μM/50 nM/6 μM) sensing system towards S^{2-} with different concentrations in Hepes buffer (pH 7.4, 20 mM). Insets: plots of fluorescence intensity of P-DNA at 582 nm versus the concentrations of MOF **1** (a), Ag^+ (b) and S^{2-} (b) respectively. Error bars represent the standard deviation for three measurements..…...………...……………………………...…………………………………S-9
Fig. S5 Fig. S5 (a) The fluorescence quenching of the P-DNA (50 nM) incubated with different concentrations of MOF **1** in different pH Hepes buffer solutions (pH = 6.5, 7.0, 7.4). (b) The fluorescence recovery of P-DNA@**1** (50 nM/9.0 μM) sensing system towards different concentrations of Ag^+ in different pH buffer solutions (pH = 6.5, 7.0, 7.4). (c) The fluorescence quenching of **1** + ds-DNA@Ag^+ (9.0 μM/50 nM/6.0 μM) sensing system towards various concentrations of S^{2-} in different pH buffer solutions (pH = 6.5, 7.0, 7.4)……….......................S-9
Reference…………………………………………………………………..…………S-10

Ag^+ and S^{2-} detection experiments

In the following experiments, all the detection systems were performed in 20 mM Hepes buffer (pH = 6.5, 7.0, 7.4) at room temperature. Both the excitation and emission slit widths are 10.0 nm. The fluorescence intensity at 582 nm (λ_{ex} = 560 nm) was used for quantitative analysis. Each experiment was carried out three times, and the mean values were taken.

First, set up the Ag^+ sensor. The solution of P-DNA (50 nM) was stirred with the increasing concentration of MOF **1** which contained 50 nM P-DNA until quenching to saturation creating P-DNA@**1** complex (Ag^+ sensor). The corresponding fluorescence spectra were measured and the quenching efficiency (Q_E, %) was calculated according to Eq. (1).

$$Q_E = (1 - F_M/F_0) \times 100\% \qquad (1)$$

Here, F_M and F_0 are fluorescent intensities at 582 nm in the presence and absence of MOF **1**, respectively.

Second, evaluate the detection sensitivity of the Ag^+ sensor and build S^{2-} sensor. Adding Ag^+ of various concentrations to the above P-DNA@**1** system, followed by incubating for 5 min to form the mixture of **1** + P-DNA@Ag^+ (S^{2-} sensor) and the fluorescence recovery efficiency (R_E) was calculated according to Eq. (2).

$$R_E = F_T/F_M - 1 \qquad (2)$$

Here F_T and F_M are the fluorescence intensities at 582 nm in the presence and the absence of Ag^+, respectively.

Third, assess the detection sensitivity of the constructed S^{2-} sensor. Adding S^{2-} of different concentrations to the above **1** + P-DNA@Ag^+ solution until quenching was saturated and the quenching efficiency (Q_E, %) was calculated according to Eq. (1).

To evaluate the selectivity of the Ag^+ and S^{2-} sensor, other metal ions (Hg^{2+}, Ba^{2+}, Ca^{2+}, Cd^{2+}, Cu^{2+}, K^+, Mg^{2+}, Mn^{2+}, Na^+, Ni^+, Pb^{2+}, Zn^{2+}, Cr^{3+}, Co^{2+}, Fe^{2+}) and anions

(SO_4^{2-}, CO_3^{2-}, NO_3^-, OH^-, HSO_4^-, $H_2PO_4^-$, F^-, Cl^-, Br^-, I^-) with the concentrations of 5-fold higher than Ag^+ and S^{2-} were investigated under the same experimental conditions.

Computational molecular simulation studies

The 3D structure of MOF **1**, P-DNA and ds-DNA@Ag^+ were constructed using Molecular Operating Environment (MOE) package.[1] The initial structure of P-DNA@**1** or **1** + ds-DNA@Ag^+ was manually built by the placement of P-DNA or ds-DNA@Ag^+ in the location 2 Å to the MOF **1** plane. Structures were first optimized in MOE using MMFF94x force field and then re-optimized in UFF of Gaussian 09[2] where Gibbs free energy calculations were simplified by calculating single point energies. Finally, Python molecule (PyMOL)[3] was employed for visual analysis of binding modes. The binding free energy difference ($\Delta\Delta G$) between reactions of MOF **1** with single chain P-DNA ($\Delta G_{P\text{-}DNA@MOF}$) or double chain ds-DNA@Ag^+ ($\Delta G_{MOF+ds\text{-}DNA@Ag^+}$) is evaluated according to the following Eq. (3).

$$\Delta\Delta G = \Delta G_{P\text{-}DNA@MOF} - \Delta G_{MOF+ds\text{-}DNA@Ag^+}$$

$$= [G_{P\text{-}DNA@MOF} - (G_{MOF} + G_{P\text{-}DNA}) - [G_{MOF+ds\text{-}DNA@Ag^+} - (G_{MOF} + G_{ds\text{-}DNA@Ag^+})]$$

$$= (G_{P\text{-}DNA@MOF} - G_{MOF+ds\text{-}DNA@Ag^+}] - (G_{P\text{-}DNA} - G_{ds\text{-}DNA@Ag^+}) \qquad (3)$$

Table S1 Crystallographic data for **1**

Formula	$C_{27}H_{21}N_3O_6Cu$	Formula weight	547.01
Crystal system	monoclinic	Space group	*C2/c*
a (Å)	31.299(3)	*b* (Å)	11.9822(9)
c (Å)	19.7406(16)	α (°)	90.00
β (°)	122.8270(13)	γ (°)	90.00
V (Å^3)	6221.1(9)	*Z*	8
T/K	291(2)	D_{calc} (g cm^{-3})	1.168
λ (Mo-K*α*) (Å)	0.71073	*μ* (cm^{-1})	0.740
Total reflections	19649	Unique reflections	6316
No. Observations	5371	No. Parameters	334
R^a	0.0376	wR^b	0.1169
GOF^c	1.110	$\Delta\rho_{max}$ (e Å^{-3})	0.689
$\Delta\rho_{min}$ (e Å^{-3})	−0.496		

^{a} $R_1 = \Sigma||F_o| - |F_c||/\Sigma|F_o|$, $wR_2 = \{\Sigma[w(F_o^2 - F_c^2)^2]/\Sigma[w(F_o^2)^2]\}^{1/2}$, GOF = $\{\Sigma[w(F_o^2 - F_c^2)^2]/(n - p)\}^{1/2}$, where n is the number of reflections and p is total number of parameters refined.

Table S2 Selected bond distances (Å) and angles (º) for MOF **1**.

bond distances [Å]			
Cu(1)-O(1)	1.9506(12)	Cu(1)-O(4)#1	1.9930(13)
Cu(1)-N(2)	2.0322(17)	Cu(1)-N(3)#2	2.0381(17)
Cu(1)-O(2)#3	2.2056(13)		
**bond angles [º]**			
O(1)-Cu(1)-O(4)#1	145.13(6)	O(1)-Cu(1)-N(2)	90.55(6)
O(4)#1-Cu(1)-N(2)	86.95(7)	O(1)-Cu(1)-N(3)#2	95.03(6)
O(4)#1-Cu(1)-N(3)#2	90.50(6)	N(2)-Cu(1)-N(3)#2	173.35(6)
O(1)-Cu(1)-O(2)#3	123.12(6)	O(4)#1-Cu(1)-O(2)#3	91.59(6)
N(2)-Cu(1)-O(2)#3	88.23(6)	N(3)#2-Cu(1)-O(2)#3	85.71(6)

Symmetry transformations used to generate equivalent atoms: #1: x, $-y$, $z - 1/2$; #2 $x + 1/2$, $-y - 1/2$, $z + 1/2$; #3: $-x + 1/2$, $-y - 1/2$, $-z + 1$.

Table S3 The analytical performance of various Ag^+ sensors

Sensor	Linear range(μM)	Detection limit (nM)	Reference
Tetraphenyl ethylene	0.5−80	874	4
Carbon dots	0−90	320	5
Iminazobe derivatixes	0−0.9	101	6
Gold nanoparticle	0.1−0.9	7.3	7
g-C_3N_4 nanosheets	0−0.04	4.2	8
P-DNA@MOF	0−1.6	3.8	This work

Table S4 Comparison of different sensing platforms for S^{2-} detection

Sensor	Linear range(μM)	Detection limit (nM)	Reference
Nanocomposite	2.67−596	138	9
gold nanoparticles	0.5−10	80	10
DNA@ copper nanoparticles	0.2−20	80	11
nanoAg–carbon	0.05−100	27	12
g-C_3N_4 nanosheets	0−0.03	3.5	8
P-DNA@MOF	0−6	5.5	This work

Table S5 The single point energy results of P-DNA, P-DNA@**1**, ds-DNA@Ag^+ and **1** + ds-DNA@Ag^+.

System	Energy (kcal/mol)	Energy (kcal/mol)
P-DNA	13.5833	8523.64
P-DNA@**1**	12.5881	7899.08
ds-DNA@Ag^+	11.8942	7463.72
**1** + ds-DNA@Ag^+	11.1862	7019.44
ΔΔG	−0.2872	-180.24

Table S6 Detection of Ag^+ in environmental water samples

Sample	C_{Ag+} in the sample (μM)	Spiked (μM)	Found (μM)	Recovery (%)	RSD (%)
Tap water	0	0.60	0.59	98.2	0.16
Lake water	0	0.60	0.61	101.8	0.98
Mineral water	0	0.60	0.61	101.6	0.58

Table S7 Detection of S^{2-} in environmental water samples

Sample	$C_{S}{}^{2-}$ in the sample (μM)	Spiked (μM)	Found (μM)	Recovery (%)	RSD (%)
Tap water	0	0.60	0.64	107.3	1.37
Lake water	0	0.60	0.59	99.0	3.10
Mineral water	0	0.60	0.61	101.2	2.57

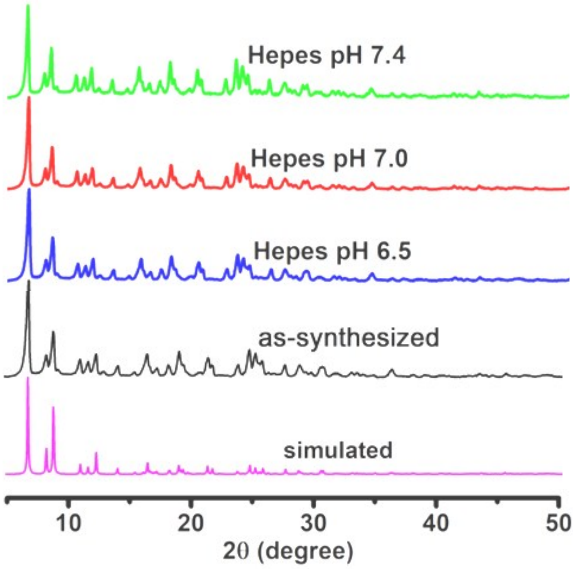

Fig. S1 PXRD patterns of MOF **1** showing an agreement among the simulated, as-synthesized and fresh powder of MOF **1** immerse in Hepes buffer (20 mM, pH = 6.5, 7.0, 7.4) for 24 h, respectively.

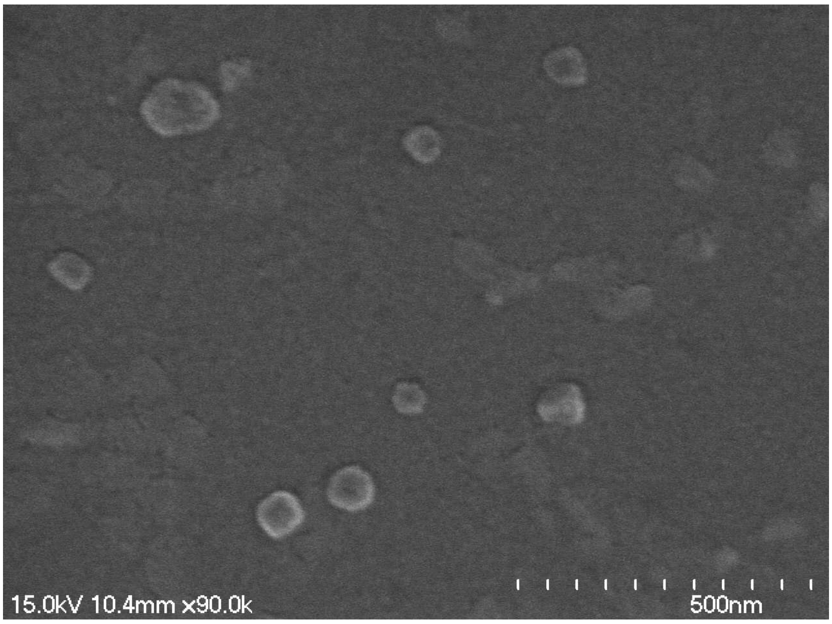

Fig. S2 The SEM image of MOF **1**.

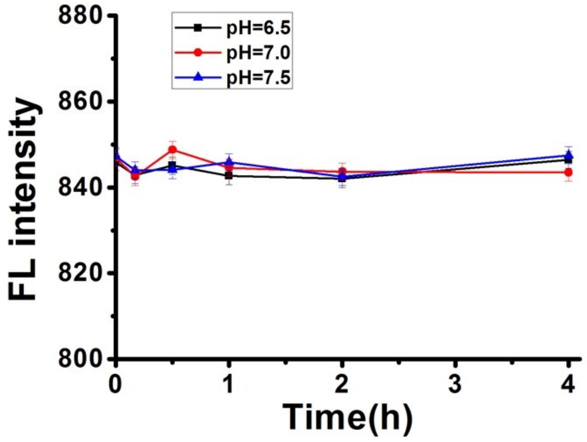

Fig. S3 Comparison of the intensity of the emission peak (582 nm) of the P-DNA in 20 mM Hepes buffer (pH = 6.5, 7.0, 7.4) for 4 h.

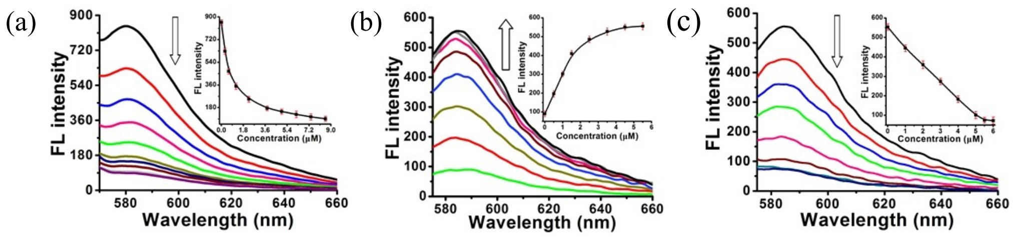

Fig. S4 (a) The fluorescence quenching of the P-DNA (50 nM) incubated with MOF **1** with increasing concentrations in Hepes buffer (pH 7.4, 20 mM). (b) The fluorescence recovery of P-DNA@**1** (50 nM/ 9 μM) sensor towards Ag^{+} with different concentrations in Hepes buffer (pH 7.4, 20 mM). (c) The fluorescence quenching of **1** + ds-DNA@Ag^{+} (9 μM/50 nM/6 μM) sensing system towards S^{2-} with different concentrations in Hepes buffer (pH 7.4, 20 mM). Insets: plots of fluorescence intensity of P-DNA at 582 nm versus the concentrations of MOF **1** (a), Ag^{+} (b) and S^{2-} (b) respectively. Error bars represent the standard deviation for three measurements.

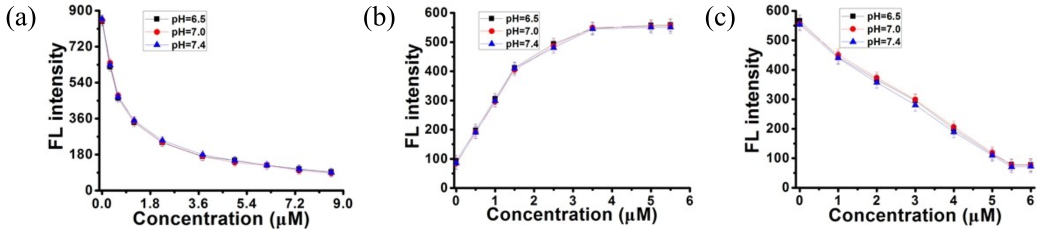

Fig. S5 (a) The fluorescence quenching of the P-DNA (50 nM) incubated with different concentrations of MOF **1** in different pH Hepes buffer solutions (pH = 6.5, 7.0, 7.4). (b) The fluorescence recovery of P-DNA@**1** (50 nM/9.0 μM) sensing system towards different concentrations of Ag^{+} in different pH buffer solutions (pH = 6.5, 7.0, 7.4). (c) The fluorescence quenching of **1** + ds-DNA@Ag^{+} (9.0 μM/50 nM/6.0 μM) sensing system towards various concentrations of S^{2-} in different pH buffer solutions (pH = 6.5, 7.0, 7.4).

Reference

1. *Molecular Operating Environment (MOE)*, 2014.09; Chemical Computing Group Inc., 1010 Sherbooke St. West, Suite #910, Montreal, QC, Canada, H3A 2R7, **2014**.
2. Frisch, M. J.; Trucks, G. W.; Schlegel, H. B.; Scuseria, G. E.; Robb, M. A.; Cheeseman, J. R.; Scalmani, G.; Barone, V.; Mennucci, B.; Petersson, G. A.; Nakatsuji, H.; Caricato, M.; Li, X.; Hratchian, H. P.; Izmaylov, A. F.; Bloino, J.; Zheng, G.; Sonnenberg, J. L.; Hada, M.; Ehara, M.; Toyota, K.; Fukuda, R.; Hasegawa, J.; Ishida, M.; Nakajima, T.; Honda, Y.; Kitao, O.; Nakai, H.; Vreven, T.; Montgomery, J. A.; Jr.; Peralta, J. E.; Ogliaro, F.; Bearpark, M.; Heyd, J. J.; Brothers, E.; Kudin, K. N.; Staroverov, V. N.; Kobayashi, R.; Normand, J.; Raghavachari, K.; Rendell, A.; Burant, J. C.; Iyengar, S. S.; Tomasi, J.; Cossi, M.; Rega, N.; Millam, J. M.; Klene, M.; Knox, J. E.; Cross, J. B.; Bakken, V.; Adamo, C.; Jaramillo, J.; Gomperts, R.; Stratmann, R. E.; Yazyev, O.; Austin, A. J.; Cammi, R.; Pomelli, C.; Ochterski, J. W.; Martin, R. L.; Morokuma, K.; Zakrzewski, V. G.; Voth, G. A.; Salvador, P.; Dannenberg, J. J.; Dapprich, S.; Daniels, A. D.; Farkas, O.; Foresman, J. B.; Ortiz, J. V.; Cioslowski, J.; Fox. D. J. *Gaussian 09*, revision D.01; Gaussian, Inc.: Wallingford CT, **2013**.
3. Schrödinger, LLC. The PyMOL molecular graphics system, version 1.8, **2015**.
4. Y. Li, H. Yu, G. Shao, F. J. Gan, *J. Photochem. Photobiol. A*, 2015, **301**, 14.
5. X. H. Gao, Y. Z. Lu, R. Z. Zhang, S. J. He, J. Ju, M. M. Liu, L. Li, W. Chen, *J. Mater. Chem. C,* 2015, **3**, 2302.
6. B. Zhao, Y. Xu, Y. Fang, L. Y. Wang, Q. G. Deng, *Tetrahedron Lett.*, 2015, **56**, 2460.
7. J. J. Du, H. Du, H. Y. Ge, *Sens. Actuators, B*, 2017, **4005**, 31456.
8. S. Wang, D. Du, M. Yang, Q. Lu, R. Ye, X. Yan, *Talanta*, 2017, **168**, 168.
9. T. Zhou, N. Wang, C. Li, H. Yuan, D. Xiao, *Anal. Chem.*, 2010, **82**, 1705.
10. H. H. Deng, S. H. Weng, S. L. Huang, L. N. Zhang, A. L. Liu, X. H. Lin, *Anal. Chim. Acta*, 2014, **852**, 218.
11. J. Liu, J. H, Chen, Z. Y. Fang, *Analyst*, 2012, **137**, 5502.
12. Z. X. Wang, C. L. Zheng, Q. L. Li, S. N. Ding, *Analyst*, 2014, **139**, 1751.
